# Immunomodulatory Effects of Four *Leishmania infantum* Potentially Excreted/Secreted Proteins on Human Dendritic Cells Differentiation and Maturation

**DOI:** 10.1371/journal.pone.0143063

**Published:** 2015-11-18

**Authors:** Wafa Markikou-Ouni, Sima Drini, Narges Bahi-Jaber, Mehdi Chenik, Amel Meddeb-Garnaoui

**Affiliations:** 1 Laboratory of Medical Parasitology, Biotechnology and Biomolecules, Institut Pasteur de Tunis, Tunis, Tunisia; 2 Unité de Parasitologie moléculaire et Signalisation, Institut Pasteur, Paris, France; 3 UPSP EGEAL Institut Polytechnique LaSalle Beauvais, Beauvais, France; INSERM, FRANCE

## Abstract

*Leishmania* parasites and some molecules they secrete are known to modulate innate immune responses through effects on dendritic cells (DCs) and macrophages. Here, we characterized four *Leishmania infantum* potentially excreted/secreted recombinant proteins (LipESP) identified in our laboratory: Elongation Factor 1 alpha (LiEF-1α), a proteasome regulatory ATPase (LiAAA-ATPase) and two novel proteins with unknown functions, which we termed LiP15 and LiP23, by investigating their effect on *in vitro* differentiation and maturation of human DCs and on cytokine production by DCs and monocytes. During DCs differentiation, LipESP led to a significant decrease in CD1a. LiP23 and LiEF-1α, induced a decrease of HLA-DR and an increase of CD86 surface expression, respectively. During maturation, an up-regulation of HLA-DR and CD80 was found in response to LiP15, LiP23 and LiAAA-ATPase, while an increase of CD40 expression was only observed in response to LiP15. All LipESP induced an over-expression of CD86 with significant differences between proteins. These proteins also induced significant IL-12p70 levels in immature DCs but not in monocytes. The LipESP-induced IL-12p70 production was significantly enhanced by a co-treatment with IFN-γ in both cell populations. TNF-α and IL-10 were induced in DCs and monocytes with higher levels observed for LiP15 and LiAAA-ATPase. However, LPS-induced cytokine production during DC maturation or in monocyte cultures was significantly down regulated by LipESP co-treatment. Our findings suggest that LipESP strongly interfere with DCs differentiation suggesting a possible involvement in mechanisms established by the parasite for its survival. These proteins also induce DCs maturation by up-regulating several costimulatory molecules and by inducing the production of proinflammatory cytokines, which is a prerequisite for T cell activation. However, the reduced ability of LipESP-stimulated DCs and monocytes to respond to lipopolysaccharide (LPS) that can be observed during human leishmaniasis, suggests that under certain circumstances LipESP may play a role in disease progression.

## Introduction

Leishmaniasis is a heterogeneous group of diseases caused by an intracellular protozoan parasite of the *Leishmania* genus, transmitted by a sandfly vector and associated with considerable morbidity and mortality throughout the world [1]. Depending on the parasite species and the host immunological response, infection with *Leishmania* results in a spectrum of disease manifestations ranging from self-healing cutaneous lesions to fatal visceral disease. After inoculation of infective metacyclic promastigotes into the dermis of a mammalian host, *Leishmania* parasites preferentially infect macrophages and DCs, both being major antigen presenting cells (APCs). While macrophages are the main host cell for *Leishmania* parasites and the main effector cells able to destroy them, DCs play a critical role in the initiation and differentiation of the adaptative immune responses to parasites leading to the control of infection or progression of disease [2–4]. To escape from the innate immune response, parasites have evolved subversion mechanisms that allow them to survive and grow inside phagocytic cells. Among these mechanisms, the inhibition of protective cytokines production, interference with effective antigen presentation, or with host cell signaling events that lead to the generation of effectors molecules and activation/deactivation of DCs and macrophages functions by parasite factors [2, 3, 5–7]. Some *Leishmania* excreted/secreted molecules are key mediators of the host-parasite interaction and are involved in these processes. Such molecules are therefore very important for parasite virulence and pathogenicity protecting the parasite from the early action of the host immune system [8]. Some of these molecules include, the secreted form of the metalloprotease GP63, the promastigote surface antigen-2 (PSA-2), the secreted acid phosphatase (sAcP), the kinetoplast membrane protein-11 (KMP-11), heat shock protein HSP-70 and cysteines proteases [9–13]. These proteins are involved in parasite survival, attachment of promastigotes to the macrophages, inhibition of antigen presentation resulting in reduced T cell activation and modulation of a number of host cell signaling molecules including blocking protein kinase C signaling, activation of protein tyrosine phosphatases and inactivation of transcription factors resulting in inhibition of cytokine production and microbicidal functions [14–19]. Most of these studies describing the interactions between *Leishmania* molecules and cells of the innate immune system were reported for macrophages but very little is known about the involvement of such molecules in modulating DCs functions. It has been shown that products secreted by *Leishmania* (*L*.) *major* promastigotes inhibit murine splenic DCs motility [20]. More recently, modulation of DCs phenotype and cytokine secretion by excreted/secreted antigens from *L*. *major and L*. *donovani* has been reported [21]. Molecules excreted and secreted by *Leishmania* parasites have been targets of interest for decades. Several studies have shown that these molecules play important roles in the infection process and modulation of local and systemic host immune factors, and that some of them such as PSA-2, KMP-11 and cysteine proteinases could also be a source of vaccine antigens against leishmaniasis [9, 22–29]. In order to identify *Leishmania* excreted/secreted proteins, we have previously used antibodies generated against promastigote culture supernatants to screen a *L*. *major* cDNA library allowing the isolation of different clones [9]. Among all the proteins revealed by sequence analysis, we have selected four and identified their orthologues in *L*. *infantum* using BLAST searches. LinJ.17.0090 encoded for EF-1α which plays an essential role in protein biosynthesis [30]. It was described as an Src homology domain containing tyrosine phosphatase (SHP-1) binding protein and SHP-1 activator and was proposed as a virulence factor since it was associated with macrophage deactivation [30–33]. In addition, a phosphoproteomic analysis of differentiating *L*. *donovani* parasites has shown that EF-1α has been identified in both promastigotes and amastigotes stages [34]. Furthermore, based on peptide quantification, a *Leishmania* exosome analysis has revealed the presence of EF-1α [35]. LinJ.13.0990 encoded for a putative protein: proteasome regulatory ATPase subunit. It showed a protein family signature: the AAA domain (ATPases Associated with a wide variety of cellular Activities). Members of the ATPase superfamily are known to be involved in essential processes of protein degradation and DNA replication by using the energy from ATP hydrolysis to remodel their respective substrates [36]. LinJ.15.0460 and LinJ.23.0070 encoded both for unknown proteins with no conserved domains. We have termed the corresponding proteins LiP15 and LiP23, respectively, in regards to their clone number identified in our previous study [9]. Here, we report a first characterization of LiEF-1α, LiAAA-ATPase, LiP15 and LiP23 based on the analysis of their immunomodulatory effects on *in vitro* differentiation and maturation of human DCs and on cytokine production by human DCs and monocytes.

## Materials and Methods

### 1. Ethic statement

Cells used in this study were obtained from peripheral blood of healthy donors as anonymously provided by the “Centre de transfusion sanguine de Tunis”. All the subjects gave their written informed consent for research purposes based on the recommendations and approval of the local ethical Committee of Institut Pasteur de Tunis (Comité d’éthique de l’Institut Pasteur de Tunis).

### 2. Production and purification of *L*. *infantum* recombinant proteins

BL21 *E*. *coli* strain cells harboring the recombinant plasmid pET-LiEF-1α, pET-LiAAA-ATPase, pET-LiP15 and pET-LiP23 were grown in LB medium, induced with 1mM isopropyl-1-thio-β-d-galactopyranoside (IPTG) for 4 h and lysed. Recombinant LiEF-1α-(His)_6_, LiAAA-ATPase-(His)_6_, LiP15-(His)_6_ and LiP23-(His)_6_ were synthesized as insoluble proteins. These proteins were solubilized in 6 M guanidine–HCl, and purified by affinity chromatography over Ni-NTA resin using an imidazole gradient elution according to the manufacturer's recommendations (GEHealthcare, Biosciences, Uppsala). The purity was demonstrated by 12% or 15% SDS-polyacrylamide gel and Coomassie blue staining (S1 Fig). Purified recombinant proteins were also tested for the presence of LPS using 10 μg/ml of polymyxin B (SIGMA–ALDRICH, Steinheim, Germany) or 100 μg/ml of proteinase K (Invitrogen) in DCs stimulated cultures. IL-10 production was strongly inhibited by proteinase K treatment whereas polymixin B did not affect this activity, indicating absence of LPS contamination in the purified recombinant proteins.

### 3. Monocytes isolation and stimulation

Human monocytes (CD14+ cell population) were isolated from peripheral blood mononuclear cells (PBMC) derived from healthy volunteers using the Ficoll Hypaque gradient (GE Healthcare Bio-Sciences AB, Sweden) method followed by positive selection using magnetic cell sorting (Midi Macs, MiltenyiBiotec, Auburn, CA, USA). Freshly purified monocytes were adjusted to 10^6^/ml in complete RPMI-1640 medium (2mM L-glutamine, 100 U/ml penicillin, 100 mg/ml streptomycin, 10% fetal bovine serum) and distributed into 96-well tissue culture plates. Cells were stimulated either by LipESP (10μg/ml P23, LiEF-1α and LiAAA-ATPase; 5μg/ml P15), in the presence or absence of LPS (Sigma-Aldrich) at 1μg/ml; then incubated for 24 h at 37°C under 5% CO_2_ or primed with 3000 U/ml of recombinant human IFN-γ (BD Biosciences Pharmingen) for 12 h then stimulated with LipESP, with or without LPS and incubated for 24 additional hours. Independent experiments were run for donor’s cells. To evaluate cytokine (IL-12p70, IL-10, TNF-α) production by monocytes, 24 h and 36 h supernatants were collected and stored at –80°C until further use.

### 4. DCs generation and stimulation

Monocytes obtained from PBMC by positive selection, were resuspended at 3.10^6^ cells/ml and cultured in complete medium at 37°C under 5% CO_2_ for 6 days. Recombinant human Granulocyte Macrophage-Colony Stimulating Factor (GM-CSF) and IL-4 (R&D Systems, Minneapolis, MN, USA) were added to culture on days 0, 2 and 4 at 1000 U/ml and 25 ng/ml, respectively. Immature DCs, harvested on day 6, were resuspended at 10^6^ cells/ml and 0,3ml of the cell suspension was plated in 24-well tissue culture plates. Independent experiments were run for donor’s cells. To induce maturation, DCs were incubated with 10 μg/ml LPS or INF-γ (10 ng/ml)/LPS for 48 h. To analyze the effect of LipESP on DCs maturation, proteins (5μg/ml LiP23 and LiEF-1α, 10μg/ml LiP15 and LiAAA-ATPase) were added to immature DCs, for 48 h and cell phenotypes (CD40, HLA-DR, CD80, CD86) and cytokines production (IL12-p70, IL-10, TNF-α) were determined. Optimal concentrations of LipESP were determined in preliminary experiments in which concentrations of 2, 5 and 10 μg/ml were tested. To analyze the effect of LipESP on LPS-induced cytokine production by DCs, LPS-stimulated cells were co-treated by LipESP for 48 h and cytokines (IL-12p70, IL-10, TNF-α) producing abilities were determined. The IL-12p70 production in response to LipESP was also evaluated after co-treatment of DCs with 10 ng/ml IFN-γ or IFN-γ/LPS for 48h. To study the effect of LipESP on DCs differentiation, monocytes were resuspended at 5x10^5^cells/ml in complete medium and plated in 24-well tissue-culture plates. Cells were incubated in the presence or absence of LipESP for 6 days. GM-CSF and IL-4 were added together with the proteins on day 0. On days 2 and 4 fresh medium was replaced with GM-CSF and IL-4 without further addition of LipESP.

### 5. Flow cytometry

After culture, immature and mature DCs were harvested for flow cytometry analysis. They were washed, resuspended at 2x10^5^/tube in PBS-1% bovine serum albumin (BSA)-0·1% NaN3 and labeled for 30 min with the appropriate concentration of fluorochrome-conjugated monoclonal antibodies to the following cell antigens: CD1a, CD40, CD86, HLA-DR, CD80, CD3, CD14 and CD19 (BD Pharmingen, San Jose, CA, USA). After two washes, cells were fixed with PBS–0,3% paraformaldehyde. Appropriate isotype controls were included. A total of 10.000 gated events, were acquired in each evaluation and analysis was performed with FACS canto II flow cytometer using DIVA software (BD Biosciences). DCs were routinely CD1a+, HLA-DR+, CD40+ and CD86+ and negative for CD14, CD3 and CD19.

### 6. Cytokine detection assays

Cytokines (IL-12p70, TNF-α and IL-10) were detected in culture supernatants using commercially available ELISA kits (BD optEIA; BD Biosciences). Recombinant cytokines were used to obtain standard curves in order to calculate cytokine concentration in the supernatants.

### 7. Statistical analysis

Results are expressed as mean ± standard deviation (SD). Statistical significance between treated and control cultures, was analyzed by Wilcoxon test (non parametric test for paired data). P-values of p< 0·05 were considered statistically significant.

## Results

### 1. LipESP down-regulate CD1a and differentially regulate HLA-DR, CD86 and CD80 surface expression on human DCs

**Fig 1:**
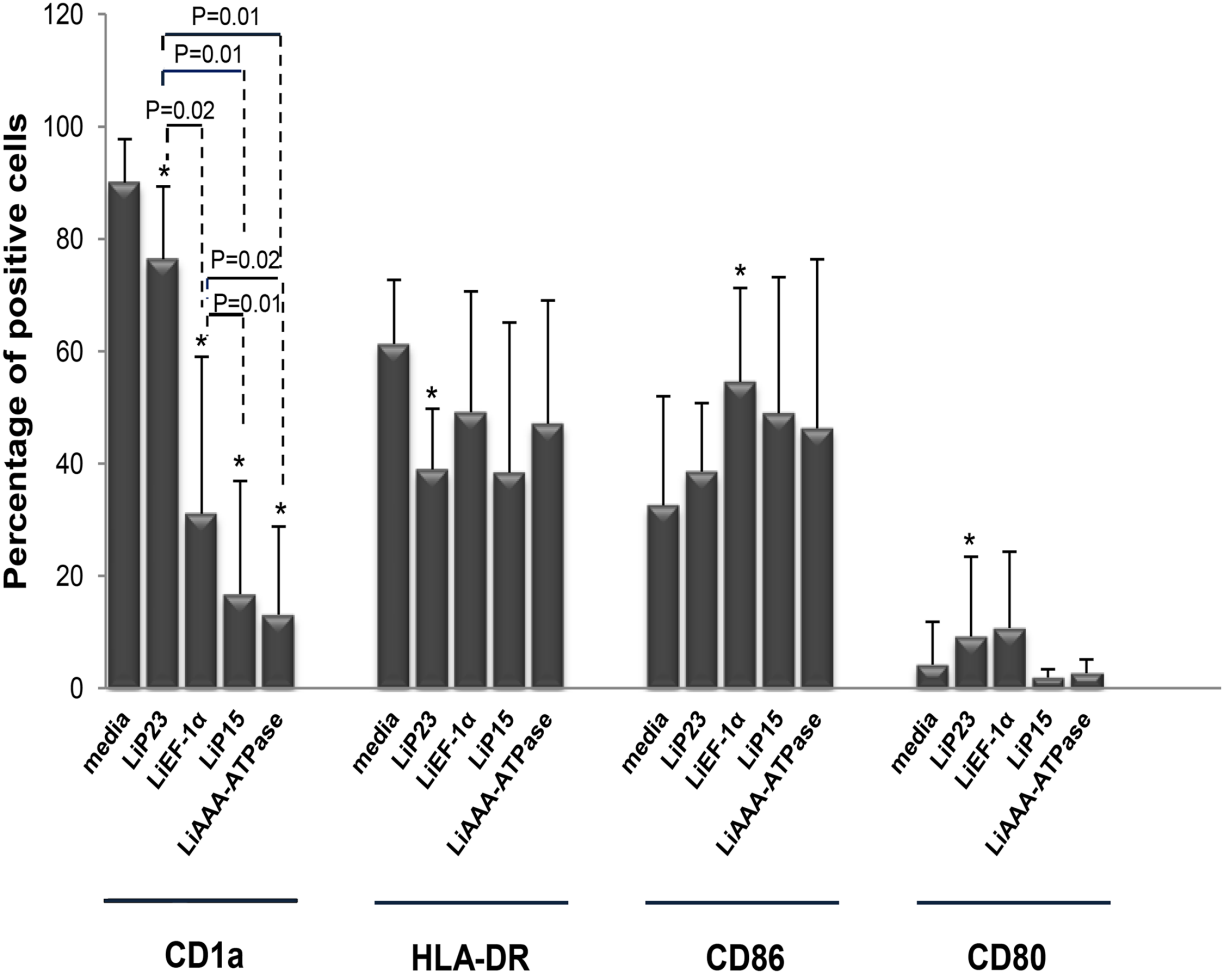
Effect of LipESP on human DCs differentiation. After 6 days of differentiation in presence or absence of LipESP, cells were harvested and labeled with appropriate antibodies. Cells with media alone represent DCs differentiation in the absence of LipESP and are considered as control cultures. Results are expressed as mean ± standard deviation of percentages of positive cells (n = 7). *: The results are statistically significant (p<0.05), when compared to control cells.

The ability of LipESP to interfere with DCs differentiation was investigated by adding proteins at the same time as GM-CSF and IL-4 to monocytes at the beginning of a 6 days culture. DCs surface expression of CD1a, HLA-DR, CD86 and CD80 molecules, was analyzed. A decrease of over 65% in the expression of CD1a was observed when DCs were differentiated in the presence of LiEF-1α, LiP15 and LiAAA-ATPase in comparison to control cultures (p≤0.02) (Fig 1). The CD1a decrease observed in response to LiP15 and LiAAA-ATPase was significantly higher than that observed for LiEF-1α (p≤0.02). An inhibitory effect of CD1a expression was also observed for LiP23 protein (p = 0.01) but was significantly lower than that observed for the other proteins (p≤0.02) (Fig 1). Furthermore, the presence of LiP23 during DCs differentiation resulted in a significant decrease in HLA-DR expression (p = 0.04) (Fig 1). We also observed an increase in CD86 and CD80 expression in presence of LiEF-1α (p = 0.02) and LiP23 (p = 0.04), respectively (Fig 1).

### 2. HLA-DR and costimulatory molecules are differentially upregulated when LipESP are added to immature DCs

**Fig 2:**
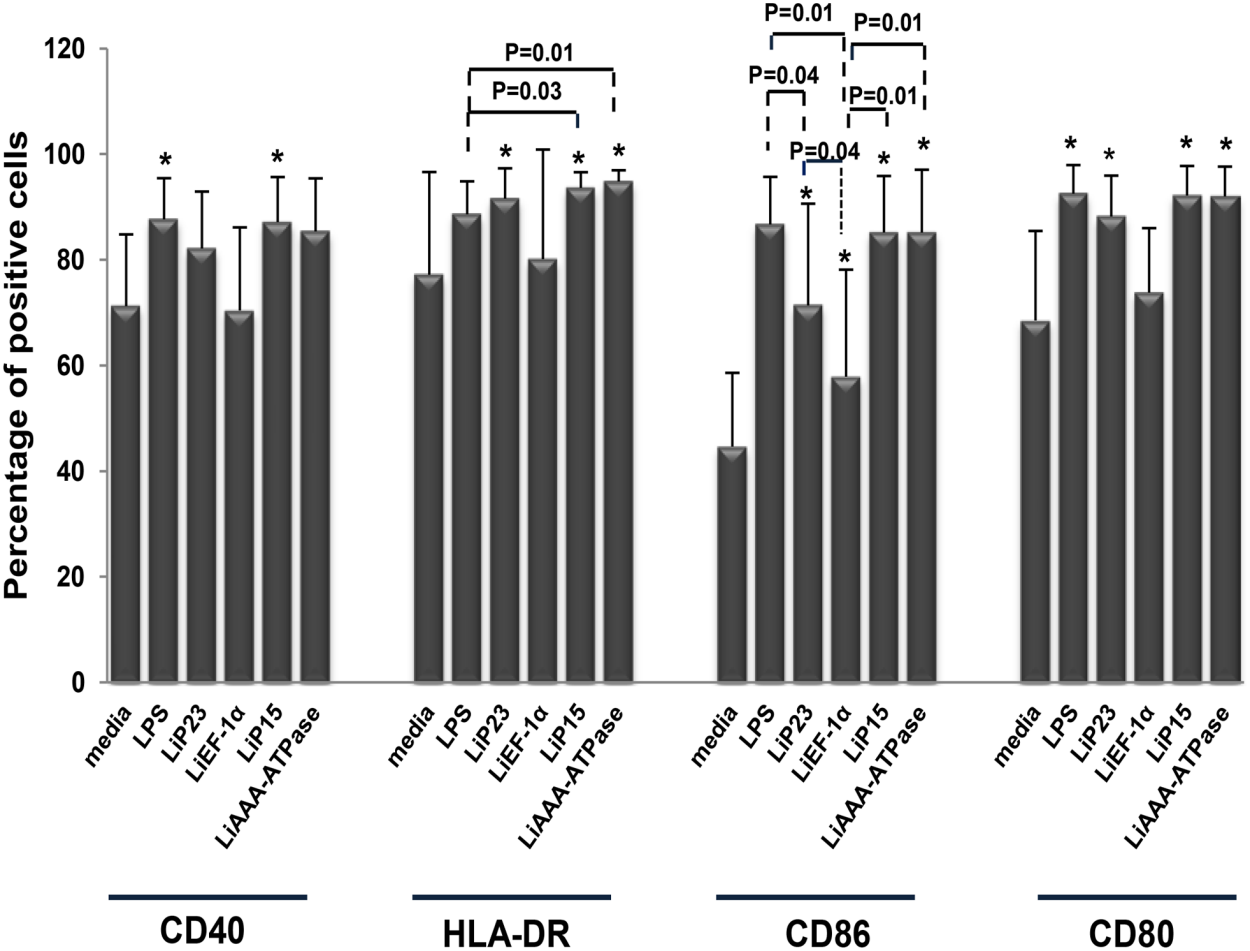
Effect of LipESP on human DCs maturation. Immature DCs were stimulated with LipESP for 48h and analysis of CD40, HLA-DR, CD86 and CD80 expression was performed by cytometry. Non-stimulated or LPS-stimulated DCs were considered as control cultures. Results are expressed as mean ± standard deviation of percentages of positive cells (n = 8). *: The results are statistically significant (p<0.05), when compared to control cells.

To investigate the effects of LipESPon DCs maturation, immature DCs were stimulated with proteins for 48 h and phenotype determinations (CD40, HLA-DR, CD86 and CD80) were performed. Results were compared to non-stimulated or LPS-stimulated DCs. LiP15 protein induced DCs maturation as shown by a significant increase of CD40, HLA-DR, CD80 and CD86 molecules when compared to non-stimulated cultures (p<0.05) (Fig 2). This result was similar to that of LPS-stimulated DCs except for the expression of HLA-DR. The significant increase in CD80, CD86 and HLA-DR expression relative to those in immature DCs showed that both LiP23 and LiAAA-ATPase were also able to induce DCs maturation (p≤0.02) (Fig 2). The LiAAA-ATPase-induced CD80 and CD86 up-regulation as well as the LiP23-induced CD80 up regulation were similar to the one observed with LPS. The two proteins also induced an increase in CD40 expression but this was not statistically significant. However, LiEF-1α protein was only able to up-regulate the costimulatory marker CD86 (p = 0.01) but did not affect the expression levels of the other markers studied (Fig 2). CD86 up-regulation induced by LiEF-1α was significantly lower than that induced by the other LipESP and by LPS.

### 3. LipESP modulate IL-12p70, IL-10 and TNF-α production by immature and mature human DCs

**Fig 3:**
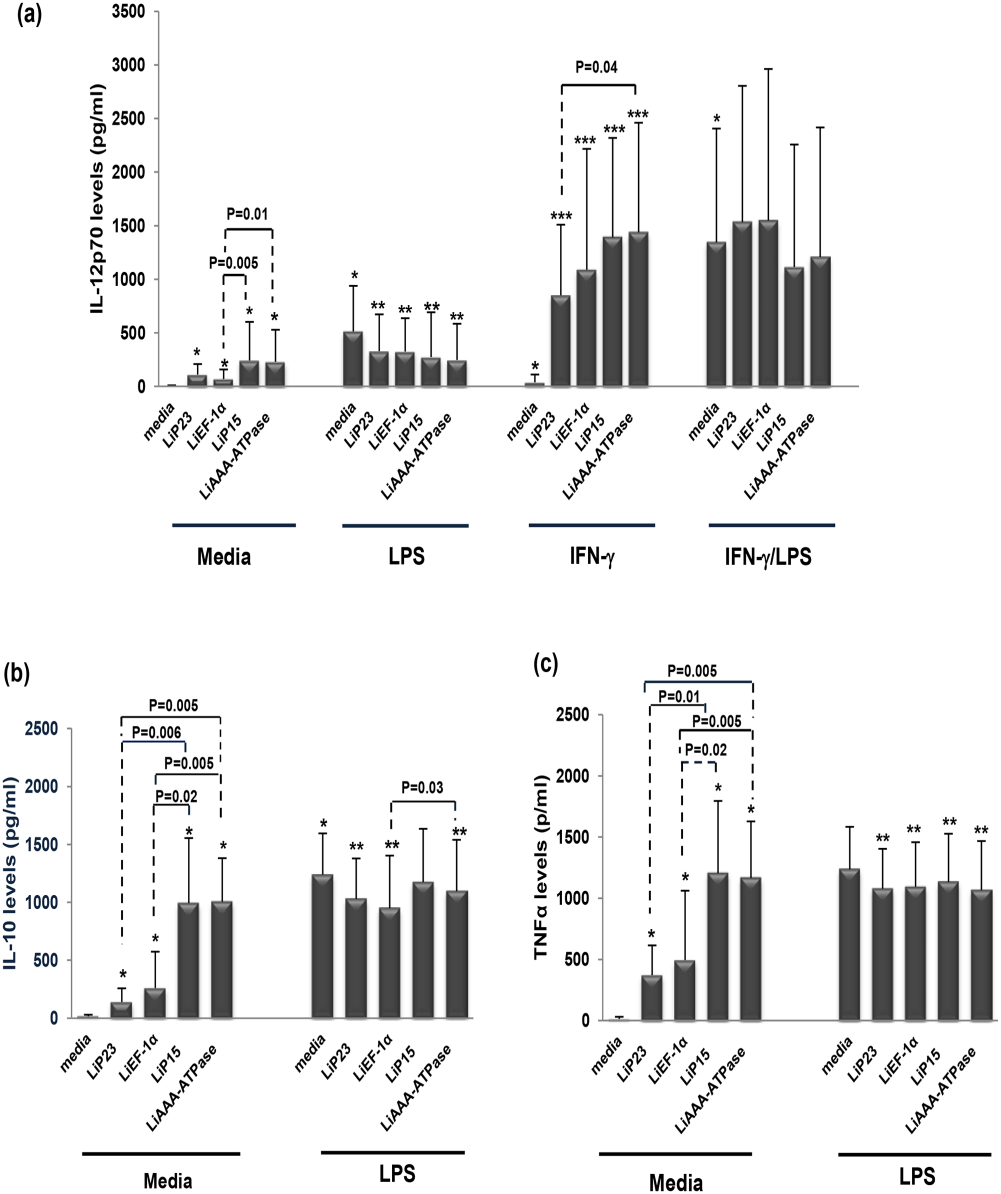
Effect of LipESP on LPS-, IFN-γ-, or IFN-γ/LPS-induced cytokine production by human DCs. Production of (a) IL-12p70, (b) IL-10 and (c) TNF-α were measured in DCs supernatant by ELISA after 48 h of stimulation by LipESP in presence or absence of LPS or IFN-γ or IFN-γ/LPS. Results are expressed as mean ± standard deviation (n = 10). * Statistically significant difference between non stimulated and LipESP-, LPS-, IFN-γ and IFN-γ/LPS-stimulated DCs (p<0·05); ** statistically significant difference between LPS-stimulated and LipESP/LPS-co-treated DCs (p<0·05); *** statistically significant difference between IFN-γ stimulated and LipESP/IFN-γ-co-treated DCs (p<0·05).

To evaluate the effect of LipESP on cytokines production by DCs, proteins were incubated with immature DCs for 48h and IL-12p70, IL-10 and TNF-α production was analyzed. All LipESP were able to induce significant levels of IL-12p70, IL-10 and TNF-α by DCs when compared with non-stimulated cells (Fig 3). However, significant differences were noted between their cytokine-inducing capacities. Indeed, LiP15 and LiAAA-ATPase induced significantly higher cytokines levels in comparison to LiP23 and LiEF-1α (p≤0.02) except for IL-12-induced by LiP23 for which we did not observe significant differences with the other proteins (Fig 3a). Analysis of LipESP effects on cytokines production during LPS-induced maturation of DCs showed that LPS-induced IL-12p70 production was down-regulated in the presence of LipESP (p≤0.02) (Fig 3a). LPS-induced IL-10 and TNF-α productions were down-regulated by LiP23, LiEF-1α and LiAAA-ATPase (p<0.05) (Fig 3b and 3c). Whereas LiP15 protein also down-regulated LPS-induced TNF-α production, it did not affect LPS-induced IL-10 production (Fig 3b and 3c). The effects of LipESP on IL-12p70 production were also analyzed during IFN-γ/LPS-induced maturation of DCs or during a co-treatment with IFN-γ. LipESP did not affect IL-12p70 production in IFN-γ/LPS matured DCs but significantly enhanced IFN-γ-induction of this cytokine (p≤0.02) (Fig 3a).

### 4. LipESP modulate IL-12p70, IL-10 and TNF-αproduction by human monocytes

**Fig 4:**
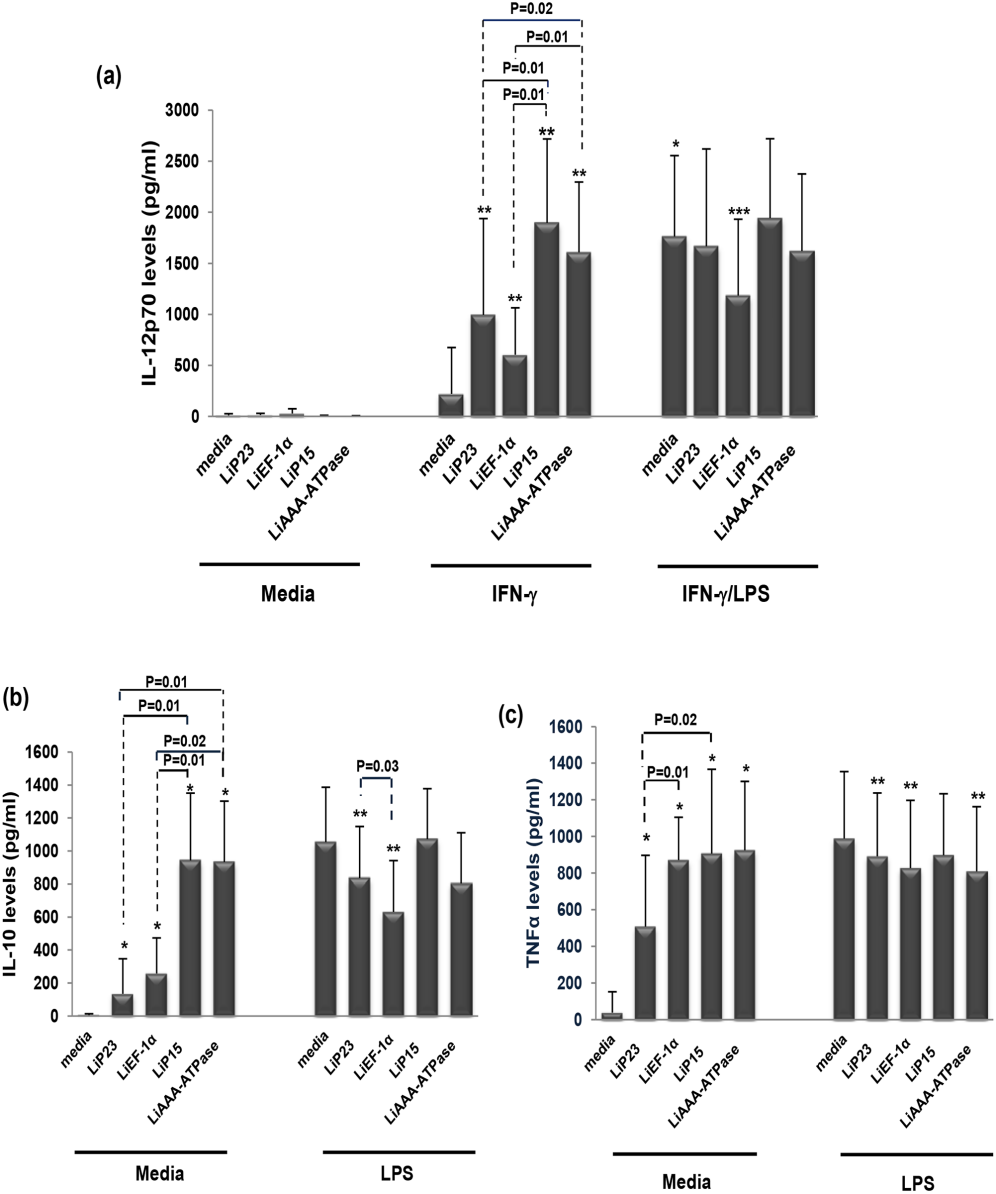
Effect of LipESP on LPS-, IFN-γ-, or IFN-γ/LPS-induced cytokine production by human monocytes. Production of (a) IL-12p70, (b) IL-10 and (c) TNF-α were measured in monocytes supernatant by ELISA after 24 h of stimulation by LipESP in presence or absence of LPS (for IL-10 and TNF-α analysis) or after 12 h of priming with IFN-γ followed by 24 additional hours of stimulation by LipESP with or without LPS co-treatment (for IL-12 analysis). Results are expressed as mean ± standard deviation (n = 9). * Statistically significant difference between non stimulated and LipESP-, LPS-, and IFN-γ/LPS-stimulated monocytes (p<0·05); ** Statistically significant difference between IFN-γ stimulated and LipESP/IFN-γ-co-treated monocytes (p<0·05) for IL-12p70 and between LPS-stimulated and LipESP-LPS-co-treated monocytes (p<0·05) for IL-10 and TNF-α; *** Statistically significant difference between IFN-γ/LPS stimulated and LipESP/IFN-γ/LPS-co-treated monocytes (p<0·05).

LipESP were used to stimulate monocytes and their effects on cytokines production by these cells co-treated or not by IFN-γ, LPS or IFN-γ/LPS were analyzed. As shown in Fig 4, monocytes were unable to produce IL-12p70 when stimulated by LipESP but significant IL-10 and TNF-α levels were observed compared to non stimulated cells (p = 0.007) (Fig 4b and 4c). LiP15 and LiAAA-ATPase -induced IL-10 levels were significantly higher compared to those induced by LiP23 and LiEF-1α (p≤0.02) (Fig 4b). LiP15 and LiEF-1α-induced TNF-α levels were higher than those induced by LiP23 (p≤0.02) (Fig 4c). To determine whether IFN-γ priming could influence the IL-12-inducing capacity of LipESP, monocytes were first primed with IFN-γ for 12 h then stimulated with proteins. A significant increase of LipESP-induced IL-12p70 production was observed as a consequence of IFN-γ priming (p = 0.01) (Fig 4a). Again, LiP15 and LiAAA-ATPase had higher inducing capacities compared to LiP23 and LiEF-1α (p≤ 0.02) (Fig 4a). We also investigated whether LipESP could affect cytokine production by LPS-treated monocytes. Monocytes were first incubated with or without IFN-γ then co-treated with LPS and proteins (to analyze IL-12p70 or IL-10 and TNF-α, respectively) (Fig 4). LiEF-1α was the only protein able to down regulate IFN-γ/LPS-induced IL-12 production by monocytes (p = 0.01) (Fig 4a). LPS-induced IL-10 was down-regulated by LiEF-1α and LiP23 (p = 0.007) with a significant difference between the two proteins. Finally, LPS-induced TNF-α production was down-regulated by LiEF-1α, LiP23 and LiAAA-ATPase (p≤ 0.02) (Fig 4b and 4c).

## Discussion

*Leishmania* excreted/secreted proteins likely play crucial roles in parasite virulence as well as host-parasite interactions, more particularly through the modulation of the host immune response [8, 12, 35, 37, 38]. In this paper, we examined the immunomodulatory effects of four potential *L*. *infantum* excreted/secreted proteins: LiEF-1α, LiAAA-ATPase, LiP15 and LiP23 on differentiation and maturation of two major phagocyte populations, DCs and monocytes playing critical roles during infection. The first aim of our study was to evaluate the ability of these proteins to interfere with DCs differentiation. We showed that LipESP were able to significantly reduce CD1a expression, with the most important effect observed for LiEF-1α, LiAAA-ATPase and LiP15. CD1a is member of the CD1 group I molecules, a family of cell surface glycoproteins that directly bind a variety of lipids and present them for T cell recognition at the surface of APCs [39, 40]. In humans three groups of CD1 isoforms have been identified, group 1 (CD1a, CD1b, CD1c), group 2 (CD1d), and group 3 (CD1e) while mice have only CD1d, making the *in vivo* analysis of group 1 CD1-restricted T cells difficult. However, the development of humanized mouse in which the human CD1 system is present and group 1 CD1 transgenic mouse models as well as the expansion of CD1-tetramer technology have facilitated the study of T cell reactivity to CD1/lipid complexes and provided evidence that group 1 CD1-restricted T cells participate in adaptive immune responses during human infection [41–43]. Most studies examining microbial antigen presentation by group 1 CD1 molecules have focused on *Mycobacterium tuberculosis* (Mtb). It was shown that group 1 CD1-restricted T cells produce IFN-γ and TNF-α upon encountering mycobacterial antigens supporting the role of these cells in protective immunity against Mtb infection [41, 44, 45]. Both mycobacterial infection and immunization with Mtb lipids elicit group 1 CD1-restricted Mtb lipid-specific T cell responses in human group 1 CD1 transgenic mice [41]. Interestingly, these cells exhibit rapid secondary responses, similar to conventional T cells suggesting that they could serve as targets for the development of novel vaccines [41]. However little is known about the involvement of group 1 CD1-restricted T cells in other microbial infection including leishmaniasis. The engagement of CD1 molecules by human T cells and functional consequences on T cell activation during *Leishmania* infection is still largely unknown. To our knowledge, no *Leishmania*-derived glycolipid antigen presented by this pathway has been identified to date in humans. However some data support the involvement of the CD1 pathway during *Leishmania* infection. Studies including ours have shown that live *L*. *major*, *L*. *donovani* [46–48] and *L*. *amazonensis* [49] promastigotes were able to significantly downregulate CD1a expression on human DCs. It was also shown that *Leishmania*-induced down-regulation of CD1 expression was not mediated by LPG or other phosphoglycans [47]. There are evidences that *Leishmania*-induced CD1 down-regulation is associated with a reduced ability of DCs to present antigen and to stimulate a CD1-restricted T cell response. Indeed, CD1 down regulation induced by *L*. *donovani* in DCs was associated with a reduced ability of DCs to stimulate an Mtb restricted T lymphocyte response [46]. A lower production of IFN-γ was observed in the supernatants of autologous cultures in which DCs differentiated in the presence of *L*. *amazonensis* parasites were used as APC [49]. These results suggest that *Leishmania*-CD1 down-regulation may be associated with a down-regulation of the Th1-adaptative immune response and therefore with the establishment of a disease-promoting immune response. However, *L*. *infantum* did not alter CD1a expression in infected DCs, but, in contrast, up-regulated CD1d cell surface expression [50]. These cells were efficiently recognized and killed by NKT cells that produce IFN-γ and a cytotoxic response which may facilitate the development of Th1 responses against *Leishmania* [50]. These contradictory results could be explained by the differences between the biology of the *Leishmania* species. Based on all these data and our results, we can suggest that through their capacity to down-regulate CD1a expression, LipESP could be associated with an impairment of CD1-restricted T cells activation by DCs. We also showed that HLA-DR expression was down regulated in LiP23-stimulated cells while CD86 expression was up regulated in LiEF-1α-stimulated cells, during DCs differentiation. Reduced levels of MHC class II but high CD86 levels were observed during DCs differentiation in the presence of live *L*. *amazonensis*, *L*. *donovani*, *L*. *major and M*. *tuberculosis* [47, 49, 51]. These changes were associated with a reduced capability to induce proliferation and IFN-γ secretion by T lymphocytes [49, 51]. Our results suggest that LiP23 and LiEF-1α, when present during DCs differentiation, could interfere with antigen presentation and optimal costimulatory activity and consequently have an impact on T cell activation, which could promote parasite survival. The second part of this study was to assess the ability of LipESP to induce DCs maturation. Stimulation of immature DCs with LiP23, LiP15 and LiAAA-ATPase resulted in a significant increase in HLA-DR, CD80, and CD86 surface expression, an indication of DCs maturation. LiP15 was the only protein also able to induce a significant up-regulation of CD40 expression. LiEF-1α only induced a CD86 up-regulation. Previous studies mainly using live parasites showed that both *L*. *major* and *L*. *donovani* were either able to induce up-regulation of CD80, CD86, CD40 and HLA-DR molecules [52–54] or had no effect on the expression of these molecules in immature DCs [21, 47, 52, 55, 56]. Effects of some parasite components on costimulatory molecules expression by human DCs were also reported. *Leishmania* eukaryotic initiation factor (LeIF), an exosomal protein, was able to up-regulate CD80 expression in human DCs [57]. However, phosphoglycans family of virulence-associated antigens was involved in inhibition of DCs maturation [55] whereas *Leishmania* exosomes did not alter the expression of HLA-DR, CD80, or CD86 in immature DCs [35]. Interestingly, it has been suggested that changes in the protein cargo of *Leishmania* exosomes may influence the impact of these vesicles on myeloid cell function [35]. In addition to induction of costimulatory molecule expression, we also demonstrated that LipESP activated DCs but not monocytes for a significant IL-12p70 production. The LipESP-induced IL-12p70 production was significantly enhanced by a co-treatment with IFN-γ in both cell populations. LipESP also induced significant levels of TNF-α and IL-10 in both cell populations with significantly higher cytokine-inducing capacities for LiP15 and LiAAA-ATPase. *In vitro* infection studies have mainly showed that in the absence of other stimuli, *Leishmania* parasites can trigger relatively weak or no cytokine production by DCs [21, 48, 52–54]. Similarly to our results, IL-12 production by infected DCs can be markedly enhanced by the addition of exogenous stimuli such as IFN-γ, IFN-γ/LPS, and CD40L [52–54]. LeIF, LPG and KMP-11 were described as inducing IL-12 production in DCs but not or to a lesser extent in monocytes [37, 38, 57, 58]. NF-kB was involved in the differential production of IL-12 between DCs and macrophages [38, 58, 59]. Recently, it was suggested that the regulation of type I IFN-associated signaling pathways was involved in *L*. *major*-induced expression of IL-12 in DCs [60]. *Leishmania*-induced IL-12 and TNF-α in matured DCs, was associated with the capacity of these cells to induce Th1 responses and IFN-γ production which are critical for resistance and cure of leishmaniasis [35, 38, 52, 53]. *Leishmania* promastigotes or amastigotes infected DCs, were able to induce a Th1 response with IFN-γ production by autologous T lymphocytes from leishmaniasis patients [52, 53]. *Leishmania* HSP100-/- exosomes promoted the differentiation of naïve CD4 lymphocytes into Th1 cells [35]. More recently, presentation of KMP-11 antigen by DCs to autologous T cells from visceral leishmaniasis patients resulted in a significant IFN-γ production by CD4+ T cells [38]. Our results showed that among LipESP, LiP15 and LiAAA-ATPase were the most efficient to induce DCs maturation and suggest that these proteins could be involved in T lymphocyte activation and IFN-γ production upon antigen presentation by DCs. However, LiEF-1α only induced a weak up-regulation of CD86 and cytokine production suggesting partial DCs maturation. Interestingly, partially matured DCs conditioned by inflammatory mediators or low concentrations of TLR ligands have been shown to instruct Th2-cell responses. It has been shown that partially matured DCs injected into mice before *L*. *major* infection were associated to the development of a Th2 response whereas fully matured DCs induced a Th1 response, suggesting that the differentiation stage of DCs determines Th1/Th2 differentiation [61]. More recently, it was demonstrated that *T*. *brucei* antigens induced partial DCs maturation that was associated with the differentiation of Th2-cell responses in vitro and in vivo [62–64]. Whether LiEF-1α is associated with the generation of a Th2 response, needs to be further investigated. LipESP also induced a significant IL-10 production in DCs and monocytes. IL-10 promotes the differentiation of tolerogenic DCs which play an important role in activating regulatory T (Treg) cells [65]. Treg cells play key roles in regulating the balance of Th1/Th2 immunity and in preventing excessive damages during the inflammatory responses and have been described during *Leishmania* infection [66, 67]. Furthermore, a recent study suggested that Treg cells are induced by *L*. *major* excreted/secreted antigens [68]. However, it seems unlikely that the LipESP-induced DCs profiles may correspond to tolerogenic DCs, except for the one induced by LiEF-1α. Indeed, tolerogenic DCs were characterized by low levels of MHC Class II and costimulatory molecule expression along with production of IL-10 and impairment of IL-12 production. Finally, we observed that LPS-induced cytokine production during DCs maturation or in monocyte cultures was significantly down regulated by LipESP co-treatment, suggesting that the presence of LipESP leads to a reduced ability to respond to inflammatory stimuli. Altered DCs responsiveness to exogenous stimuli has been reported by our group and by others in the presence of *Leishmania* antigens and live parasites [21, 35, 48, 49]. Stimulation of LPS/TNF-α matured DCs with *Leishmania* excreted/secreted antigens induced a decrease in IL-10 and IL-12p70 productions [21]. *Leishmania* exosomes containing virulence factors such as LiEF-1α inhibited cytokine production by CD40L-matured DCs and by *Leishmania*-infected or IFN-γ-treated monocytes, suggesting that exosomes are able to modulate the immune response to make it permissive for infection [35]. Considering the presence of LPS during bacterial superinfections that can be observed in leishmaniasis [69, 70], it is tempting to speculate that LipESP could benefit from the presence of LPS to immunomodulate DCs functions to the parasite advantage.

In conclusion, our results suggest that LiEF-1α, LiAAA-ATPase, LiP15 and LiP23 are among the parasite products responsible for incomplete differentiation of DCs, probably interfering with DCs development pathway and consequently inducing a less effective immune response against the parasite. However, the presence of LipESP, especially LiP15 and LiAAA-ATPase, during DCs maturation leads to their activation and this could contribute to the modulation of the inflammatory response and host resistance, suggesting that such molecules could have a great potential to be used as therapeutic agents to modulate inflammatory diseases.

## Supporting Information

S1 Fig Analysis of recombinant LiEF-1α, LiAAA-ATPase, LiP15 and LiP23 by SDS PAGE gel. Purified proteins were separated by electrophoresis in a 12% SDS-PAGE for LiEF-1α, LiAAA-ATPase and LiP15 visualization, and in a 15% SDS-PAGE for LiP23 visualization. Molecular Weight markers were marked in kDa.
(DOCX)
