# Supplementary material for: Immunomodulatory Effects of Four Leishmania infantum Potentially Excreted/Secreted Proteins on Human Dendritic Cells Differentiation and Maturation
Source: PLoS One. 2015 Nov 18;10(11):e0143063. doi: 10.1371/journal.pone.0143063 (PMC4651425; doi:10.1371/journal.pone.0143063)
Supplement: S1 Fig — Purified proteins were separated by electrophoresis in a 12% SDS-PAGE for LiEF-1α, LiAAA-ATPase and LiP15 visualization, and in a 15% SDS-PAGE for LiP23 visualization. Molecular Weight markers were marked in kDa. (DOCX) [file pone.0143063.s001.docx]

**S1 Fig. Analysis of recombinant LiEF-1α, LiAAA-ATPase, LiP15 and LiP23 by SDS PAGE gel.** Purified proteins were separated by electrophoresis in a 12% SDS-PAGE for LiEF-1α, LiAAA-ATPase and LiP15 visualization, and in a 15% SDS-PAGE for LiP23 visualization. Molecular Weight markers were marked in kDa.


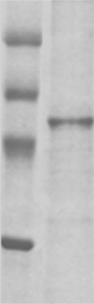


**94**

**67**

**43**

**30**


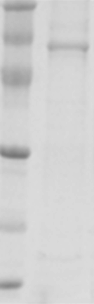


**94**

**67**

**43**

**30**

**20**

**14**


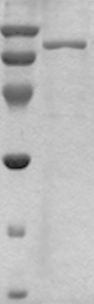


**94**

**67**

**43**

**30**

**20**

**14**


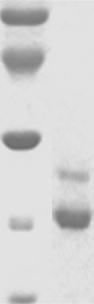


**67**

**43**

**30**

**20**

**14**

**LiEF1α**

**LiAAA-ATPase**

**LiP15**

**LiP23**

The recombinant LiEF-1α, LiAAA-ATPase, LiP15 and LiP23 were produced in BL21 E. coli cells using the pET prokaryotic expression system. Proteins were then purified by affinity chromatography over Ni-NTA resin and purity was assessed by SDS-PAGE using 12% or 15% polyacrylamide gels. Coomassie blue stained bands of 49, 49, 77 and 20 kDa corresponded to LiEF-1α, LiAAA-ATPase, LiP15 and LiP23, respectively.
